# Electrical Transport and Magnetic Properties of Metal/Metal Oxide/Metal Junctions Based on Anodized Metal Oxides

**DOI:** 10.3390/ma14092390

**Published:** 2021-05-04

**Authors:** Arkadiusz Zarzycki, Juliusz Chojenka, Marcin Perzanowski, Marta Marszalek

**Affiliations:** Institute of Nuclear Physics Polish Academy of Sciences, PL-31342 Krakow, Poland; juliusz.chojenka@ifj.edu.pl (J.C.); marcin.perzanowski@ifj.edu.pl (M.P.); marta.marszalek@ifj.edu.pl (M.M.)

**Keywords:** magnetic junction, magnetoelectric properties, metal-oxides, porous, anodization, thin film

## Abstract

In this paper, we describe magnetoelectric properties of metal/metal-oxide/metal junctions based on anodized metal oxides. Specifically, we use Ti and Fe metallic layers separated by the porous metal-oxides of iron or titanium formed by the anodization method. Thus, we prepare double junctions with at least one ferromagnetic layer and measure magnetoresistance, as well as their current-voltage and magnetic characteristics. We find that magnetoresistance depends on that junction composition and discuss the nature of differential resistance calculated from *I-V* characteristics. Our findings show that a top metallic layer and the interface between this layer and anodized oxide, where strong interatomic diffusion is expected, have the strongest influence on this observed behavior.

## 1. Introduction

There has been a growing interest in recent years in the magnetic and electrical properties of metal oxides [1,2], for example, magnetic semiconductors, dilute semiconductors (e.g., doped titanium oxides) [3,4], and not-dilute semiconductors (e.g., hematite) [5], and the magnetic half-metals, such as magnetite [6]. The main focus of our study is the junctions composed of the titanium- and iron-based metal oxides, which are widely considered for electronics and spintronics [7,8] because the semiconducting metal oxide/metal heterostructures can form a junction with the Schottky barrier [9]. Additionally if the magnetic oxide is used, it can induce spin-polarization of the charge carriers: an effect which is found in semiconducting hematite [10], insulating maghemite [11], and half-metallic magnetite [12]. Finally, metallic oxide semiconductors with a wide bandgap above 2 eV can work at elevated temperatures, high frequencies, and high powers [13].

The patterned semiconducting oxide layers are increasingly considered as a potential material for photoelectrochemical and photocatalytic purposes [14], as demonstrated in the case of titanium oxide with a bandgap of ∼3.0–3.2 eV [15]. The nanotubular anodized titanium oxide was shown to form a Schottky barrier between Ti and TiO${}_{2}$ and the importance of structural defects on semiconducting properties was further investigated recently [16,17]. The impact of defects, especially in non-stoichiometric TiO${}_{2}$, can lead to the *n*- (oxygen deficient) or *p*-type (titanium deficient) semiconductors [18]. Defects can also induce weak ferromagnetism in paramagnetic titanium oxides, as observed for anatase, rutile, and TiO rock salts [19,20,21]. Iron oxides show a wide range of magnetic and transport properties. For example, the magnetite has an inverse spinel structure and is a ferrimagnetic half-metal that transforms into insulator below the Verwey transition (∼125 K) [22]. On the other hand, the hematite (α-Fe${}_{2}$O${}_{3}$) is a semiconductor with a bandgap of 2.0 eV [23], which is antiferromagnetic below Néel temperature of 950 K and at 260 K undergoes the Morin transition between easy-axis and easy-plane antiferromagnet with small canting between sublattices resulting from Dzyaloshinskii–Moriya interaction [24]. Maghemite (γ-Fe${}_{2}$O${}_{3}$) is ferrimagnet with magnetite-like inverse spinel structure and hematite-like stoichiometry, for which the charge neutrality is achieved through the presence of Fe ions vacancies [25]. Hematite [26] and magnetite [27] have both demonstrated their applicability in oxide-based junctions with the Schottky barrier.

In this work, we used electrochemical anodization for the formation of oxide layers. This well-known technique allows the oxidation of metals, as well as the preparation of porous and patterned structures [28]. It is also a relatively easy and low-cost method which can be applied to large sample areas and complex surfaces. Anodization of a large variety of metals have been previously successfully demonstrated. This includes titanium [29] and iron [30] for which anodized titanium oxide (ATiO) and anodized iron oxide (AFeO) are created on the metal surface. These anodized oxides have amorphous structure and often exhibit poor crystallinity. The transformation of an anodized oxide into a well-defined crystallographic structure can, however, be performed by annealing at elevated temperatures. After the annealing at around 773 K, the anodized oxides are efficiently transformed into a mixture of oxides with dominant rutile phase for ATiO [31], and hematite for AFeO [32].

The anodized layers are often used for the preparation of nanopatterned structures for catalytics, photovoltaics, plasmonic, or biomedical materials [33,34,35,36], and as templates for patterned thin films [37,38] or nanowires [39]. In this paper, we propose an innovative idea of using the anodization process for the preparation of metal-oxide based junctions. We show results of magnetoelectric studies of junctions formed with Fe, Ti, and their oxides for which the properties strongly depend on the junctions composition. We observe a strong influence of top metallic layer, either ferromagnetic iron or paramagnetic titanium. For the case of ferromagnetic top metallic layer, the magnetoelectric effect is strong and dominates the transport properties. On the other hand, for the top paramagnetic layer, the semiconducting properties of the oxides become dominant and govern the electrical transport of the junction.

## 2. Materials and Methods

We prepared metal/anodized metal oxide/metal junctions with different compositions, as listed in Table 1. First, we deposited a metallic multilayer stack consisting of 50 nm of titanium adhesive layer followed by 100 nm of gold layer for electrical contact and a 300 nm layer of titanium or iron on 17 mm × 17 mm Si(001) substrate. The deposition was performed at room temperature with e-gun evaporation system (ESV4, Leybold GmbH, Cologne, Germany) in a vacuum chamber at the pressure of $10^{-5}$ mbar. The thickness of the films was controlled with the quartz detector. The 300 nm of Ti or Fe was deposited through round mask with a diameter of 10 mm positioned at the center of the substrate. The ATiO or AFeO oxide layers were formed using the anodization method. Approximately half of the titanium or iron layer thickness was oxidized. Next, the samples were covered with 50 nm of either titanium or iron and 50 nm of gold. The top gold layer provides electric contact and protection against contamination from the atmosphere. The deposition of the last metallic and gold layers was done through a cylindrical mask with a diameter of 7 mm, positioned at the center of the samples. The successive reduction of the planar size of deposited layers served to prevent short-circuiting at the edges between the layers.

The anodization was performed with a homemade two-electrode system with the platinum cathode and the metallic layer used as an anode (for more details, see Reference [40]). The parameters of anodization were the same for all samples, ensuring the formation of the porous oxide layers with similar thicknesses. The anodization was performed at room temperature for 45 min at a constant anodizing voltage of 5 V. The electrolyte composed of 0.3 wt.% NH${}_{4}$F + 1 wt. % H${}_{2}$O dissolved in C${}_{3}$H${}_{8}$O${}_{3}$ was used. The last step of sample preparation was thermal annealing at 747 K performed for 60 min in a vacuum chamber to improve the structural properties of ATiO and AFeO layers.

X-ray diffraction (XRD) measurements were performed with Panalytical XPert Pro diffractometer (Almelo, Netherlands) equipped with Cu lamp working at 40 kV and 30 mA using $K_{\alpha 1}$ wavelength. The Bragg-Brentano geometry was adopted and each sample was measured in 2θ range of 20–90 degrees. Details of the measurement protocol and equipment setup used in our laboratory can be found in Reference [41]. Morphology studies were performed with a scanning electron microscope (SEM, Tescan Vega 3, Fuveau, France) with a secondary electron detector. Magnetic properties were measured with MPMS SQUID XL magnetometer (Quantum Design, San Diego, CA, USA) on samples of approximately 3 mm × 3 mm size. The measurements were done in magnetic fields between −50 and +50 kOe applied in a parallel and a perpendicular direction to the sample surface (*H*‖*S* and *H*⊥*S*) and within a temperature range between 10 K and 300 K. The magnetization values were normalized with respect to the total volume of the deposited material.

**Figure 1:**
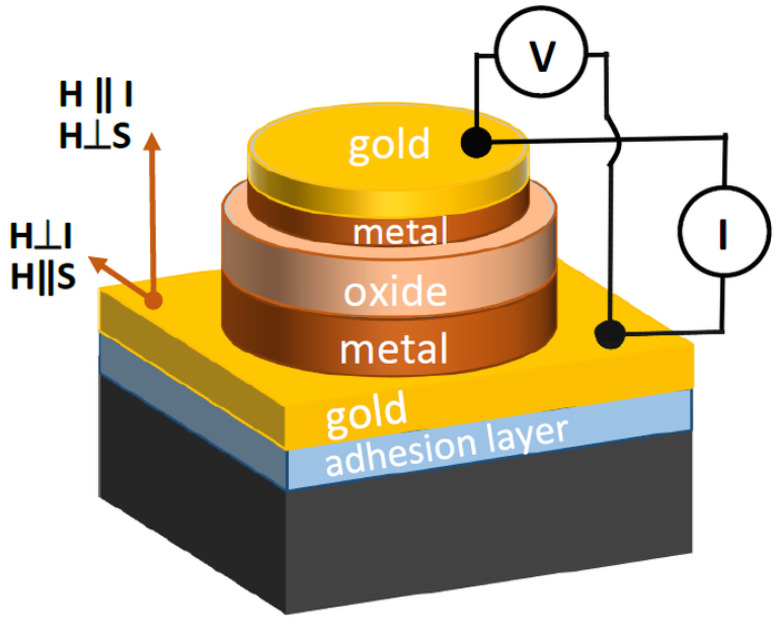
Schematic view of the metal/metal-oxide/metal junction.

The studies of electrical transport properties were performed with standard four-probe technique and involved magnetoresistance (MR) and current-voltage characteristic (*I*-*V*) measurements. A sketch of the junction is presented in Figure 1. The electrical contacts are assembled to the bottom and top gold layers and the measurement is done through the metal/metal oxide/metal junction. We used Keithley 2400 current source, Keithley 2182A nanovoltmeter, and channel switcher Keithley 3706A (Tektronix Company, Beaverton, OR, USA) during the measurements. The control of temperature and magnetic field was provided by the SQUID XL magnetometer. The MR and *I*-*V* studies were conducted in a field range of ±50 kOe at high (300 K) and low (5 K) temperatures in longitudinal and transverse geometries, i.e., *H*‖*I* (*H*⊥*S*) and *H*⊥*I* (*H*‖*S*), respectively.

## 3. Results and Discussion

### 3.1. XRD Studies

**Figure 2:**
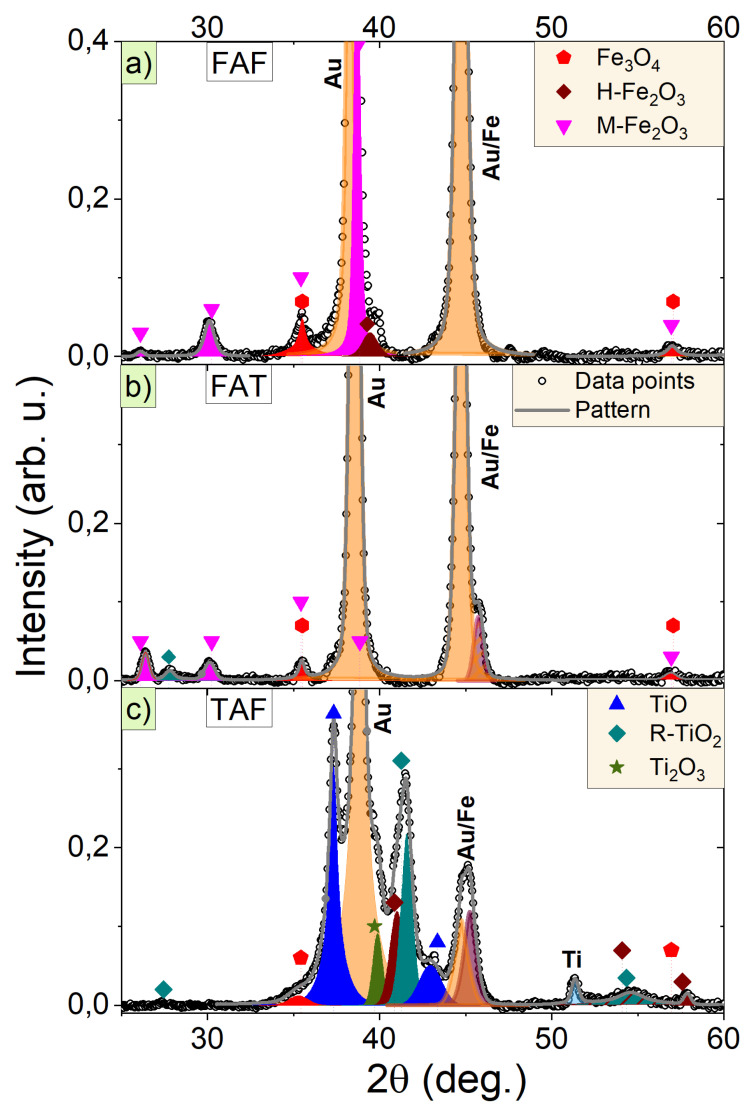
XRD patterns for (**a**) FAF, (**b**) FAT, (**c**) TAF metal/metal oxide/metal junctions. The titanium and iron oxides are marked with different tags corresponding to different metal oxides.

Diffraction patterns of FAF, FAT, and TAF junctions are presented in Figure 2. The peaks of constituent layers of Au, Ti, Fe, as well as iron and titanium oxides, are identified. The strongest signal originates from the gold layers. The peak at 38.2 degrees from Au (111) crystallographic plane with maximal intensity is observed in all junctions, showing preferential growth in this crystallographic direction, i.e., the most densely packed direction of *fcc* structure. The most intense diffraction peaks from titanium and iron layers have similar positions as gold and they overlap substantially. The presence of *bcc* Fe phase [42] is noticeable at approximately 45 degrees. The intensity of common Au/Fe peak decreases in dependence on iron content in the junction. The Ti, which typically crystallizes in *hcp* structure, can be identified only in the diffractogram of TAF junction. However, it is known that, for thin films, a stable Ti *fcc* phase can be also found with a lattice constant of a=4.05 Å [43,44], very close to the lattice constant of Au a=4.07 Å [45]. Hence, the distinction between phases of Au and *fcc* Ti from XRD studies is not possible.

The results indicate that AFeO is a mixture of hematite, magnetite and maghemite in FAF, while only magnetite and maghemite were observed for FAT junctions. The ATiO layer of TAF junction shows signal from rutile, TiO, and Ti${}_{2}$O${}_{3}$, while no anatase phase is found. In most cases, anatase is a precursor of rutile formation and for bulk the transformation of anatase into rutile takes place at temperatures of 873 K or higher [31], about 100 degrees higher than in our studies. The lack of anatase phase suggests good efficiency of the annealing process which enables the formation of a more stable rutile phase. The reduction of transformation temperature with the size of nanostructures was previously observed by Bauer et al. [46].

Furthermore, in the TAF sample, we find the diffraction maxima that can be related to hematite and magnetite. A similar but less pronounced situation is observed for FAT junction where a signal from rutile was observed. Such behavior indicates material intermixing and diffusion at the interface of anodized oxide and the top metallic layer. The interatomic diffusion happens during annealing process and results in partial oxidation of metallic layer. Such diffusion may also induce the formation of ternary iron titanates phases [47], but we have not identified corresponding diffraction maxima. The expected subtle changes in the cell parameters caused by small admixtures and small peak intensities have not been observed.

### 3.2. SEM Imaging

**Figure 3:**
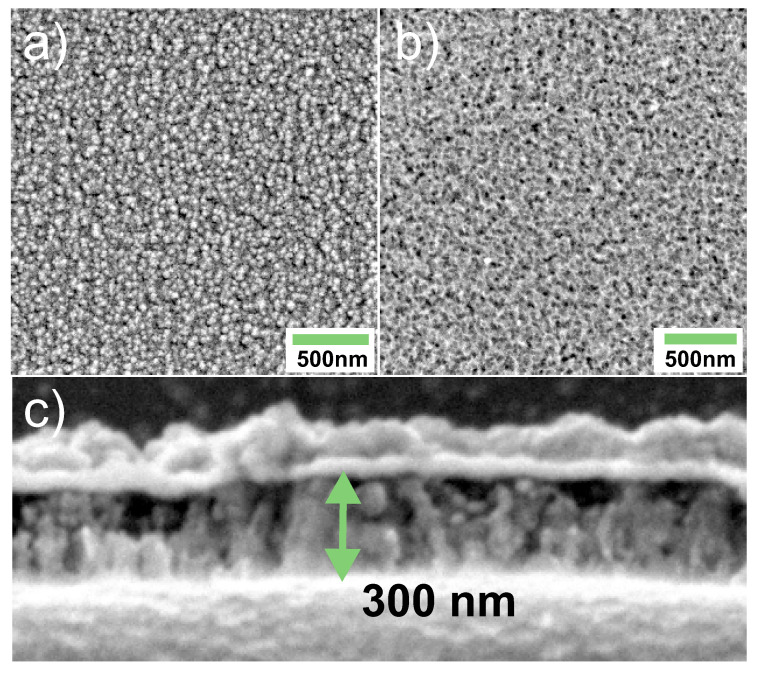
SEM images of (**a**) anodized titanium oxide layer and (**b**) anodized iron oxide layer. The bottom image (**c**) shows a cross-section of AFeO.

Figure 3 presents SEM images of the surface of ATiO and AFeO metal oxides directly after the anodization and annealing. The images were collected from the part of the sample surfaces not covered with metals, as shown in Figure 1. The morphology of titanium and iron oxide layers has a porous form with differences in grain dimensions. The size of grains observed on the ATiO surface is larger, between 75–100 nm, while, for AFeO, the grains are smaller (50 nm or less). For anodized oxide layers covered with metal, the porous pattern is replicated on the metallic surface. Figure 3c shows an illustrative cross-section of the AFeO layer. The thickness of the oxide formed after 45 min of anodization was found to be about 300 nm while the thickness of the remaining bottom metallic layer is estimated to be 150 nm, basing on anodization current versus time curves. In case of anodization process, the thickness of the oxide layer increases significantly as compared to consumed metal thickness. This process is governed by the ratio of cell volume of oxide and cell volume of metal but also depends on anodization conditions [29].

### 3.3. Magnetic Properties

**Figure 4:**
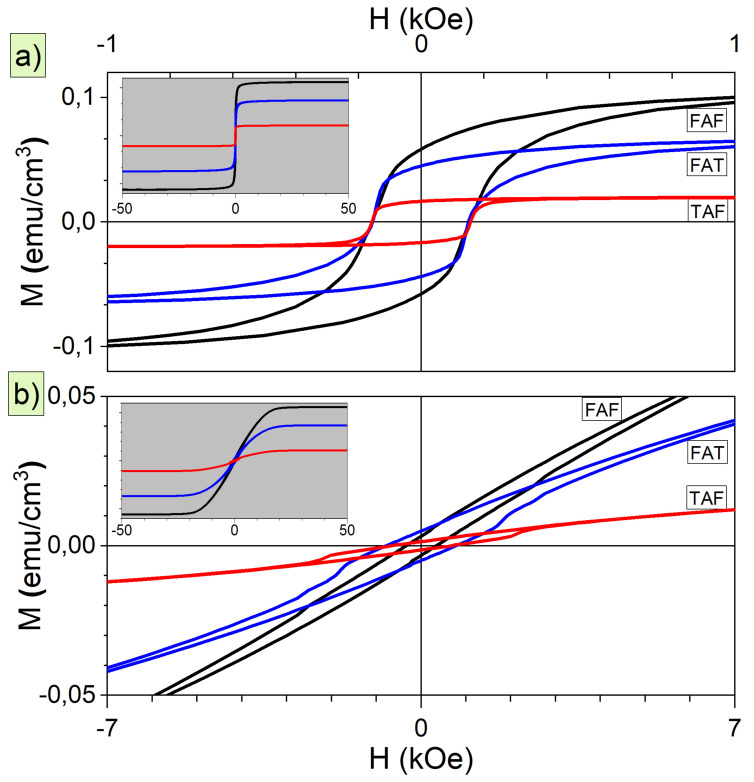
Field dependent magnetization curves for FAF, FAT, and TAF junctions measured at room temperature for (**a**) *H*‖*S* and (**b**) *H*⊥*S* geometry. The insets show the M(H) curves in the range of a magnetic field of ±50 kOe.

The magnitude of the magnetic signal strongly depends on the composition of the bottom and top metallic layers and anodized oxide layer; more iron atoms in the sample induce higher net magnetization. Figure 4 presents the results of field-dependent magnetization curves collected at room temperature for the magnetic field applied in *H*‖*S* and *H*⊥*S* directions. The insets show the results of *M*(*H*) dependencies in full range of magnetic field. The shape of the hysteresis curves indicate that an easy magnetic direction lies in the plane.

**Figure 5:**
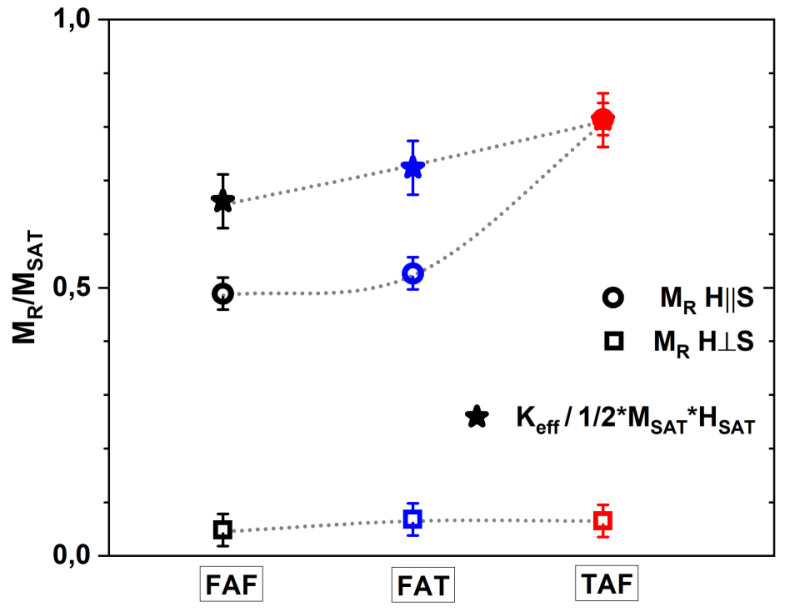
Normalized remanence magnetization ($M_{R}/M_{SAT}$) values for *H*‖*S* and *H*⊥*S* directions and normalized anisotropy constant calculated for FAF, FAT, and TAF junctions at room temperature.

The values of magnetic remanence normalized to the saturation magnetization ($M_{R}/$$M_{SAT}$) for both geometries are presented in Figure 5. The remanence values for *H*⊥*S* direction are below 0.1, while, for *H*‖*S*, geometry are around 0.5 for FAF and FAT and 0.8 for TAF junctions. Such values show strong influence of shape anisotropy associated with in-plane easy magnetic direction. We calculated, based on the approach presented in Reference [48], the effective magnetic anisotropy constant ($K_{eff}$) to confirm the role of shape anisotropy. In short, the effective magnetic anisotropy was calculated as a difference between the areas from first quadrants of easy and hard magnetic directions. To compare the values of magnetic anisotropy between the junctions, a $K_{eff}/(1/2M_{SAT}H_{SAT}$) ratio was calculated. $M_{SAT}$ and $H_{SAT}$ are the saturation magnetization and magnetic field for hard magnetic direction, and the $1/2M_{SAT}H_{SAT}$ is a maximum value of magnetic anisotropy constant [49]. Such ratio will be equal to 1 for a case of ideal magnetic anisotropy and 0 for isotropic distribution of magnetic moments. The obtained values are between 0.65–0.85 and confirm the presence of strong non-perfect in-plane magnetic anisotropy reflected also in opened curves for *H*⊥*S* direction and their slanted shape for *H*‖*S* direction. Surprisingly, the strongest magnetic anisotropy and remanence were found for TAF junction, with only one ferromagnetic layer and smaller content of iron than for FAF junction.

**Figure 6:**
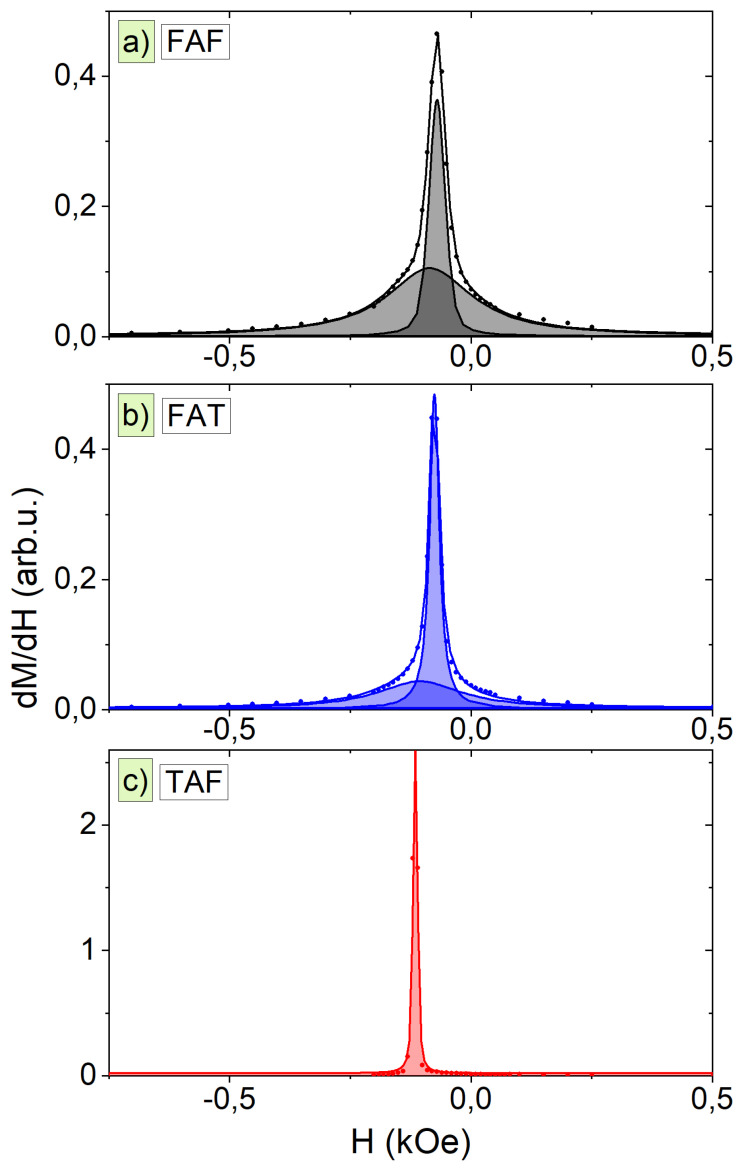
A magnetization derivative of hysteresis curves for (**a**) FAF, (**b**) FAT, and (**c**) TAF junctions measured at 300 K for *H*‖*S* geometry.

The hysteresis curves were also used to evaluate the switching field $H_{SF}$ and its distribution. The switching field, defined as the field at which the inflexion on the M(H) curve occurs corresponding to the situation when a maximal number of magnetic moments changes orientation for opposite, has been calculated as a maximum of M(H) derivative. We have also quantified the FWHM of switching field distribution SFD and percentage contributions of the magnetic components. The SFD informs about the magnetic homogeneity of the sample: if the magnetic moments alter the direction simultaneously the SFD is narrow, whereas if the switching is scattered for a wider range of magnetic fields, then the distribution of the switching field is large. Such SFD broadening can be observed for grains or clusters of magnetic material with different sizes or for chemically disordered samples. The results of the $H_{SF}$ distribution at 300 K for *H*‖*S* geometry are shown in Figure 6. The presence of two switching field components indicates the existence of two distinct magnetic contributions. These components have similar values of the mean switching field but differ in SFD widths; one is narrow, and the other is broad. The exception is TAF junction for which we found only one component with a narrow distribution. The existence of two magnetic components in FAF and FAT junctions can be explained as follows: one component with narrow distribution comes from iron layers, while the other with broad distribution originates from chemically disordered AFeO layer consisting different phases of iron oxides as was previously demonstrated by XRD studies.

The values of $H_{SF}$, SFD and coercive field ($H_{C}$) obtained at 10 K and 300 K for the magnetic field applied for easy and hard magnetic direction are collected in Table A1 of Appendix A. We observed a weak influence of temperature and the similar values of $H_{SF}$ and $H_{C}$ for *H*‖*S*, while, for hard magnetic direction, the switching field is several times larger than the coercivity. The distribution of switching fields for *H*⊥*S* geometry is very broad reflecting the influence of magnetic anisotropy. In this case, the SFD for the main component has 15–20 kOe and is comparable with $H_{SAT}$ (see Figure 4b). The remaining part of the magnetic signal that accounts for a few or several percentages for *H*⊥*S* geometry corresponds to the strongly pinned magnetic moments that are responsible for open hysteresis curves and non-zero coercivity. The pinning effect can appear at the interface between metallic film and oxide layer in this porous system.

### 3.4. Electrical Transport Properties

**Figure 7:**
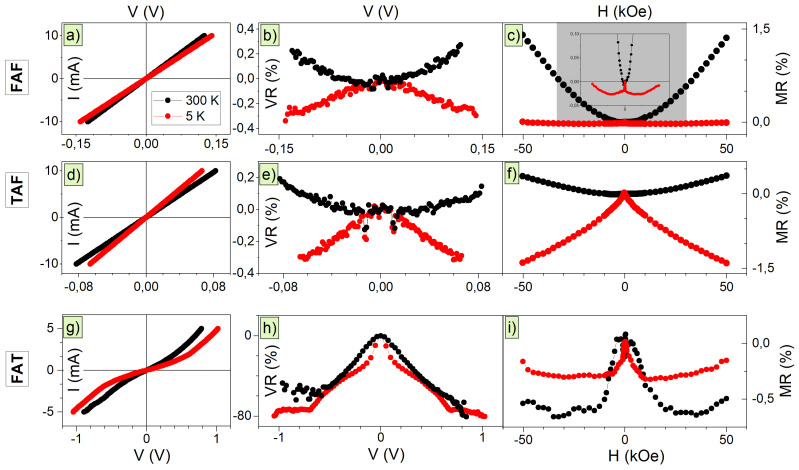
*I*-*V* characteristics (**a**,**d**,**g**) with changes of differential resistivity induced with voltage VR(V) (**b**,**e**,**h**) and magnetoresistance MR(H) (**c**,**f**,**i**) for FAF, TAF, and TAF junctions. Inset of Figure 7c is a magnification of FAF magnetoresistance dependence.

The current-voltage characteristics (*I*-*V*) and magnetoresistance dependencies $\left(MR=\frac{R(H)-R(H=0)}{R(H=0)}\right)$ of junctions were measured at 5 K and 300 K. Figure 7 presents results for longitudinal configuration since the measurement geometry does not affect the obtained results. The *I*-*V* characteristics presented in Figure 7 for FAF and TAF samples, i.e., junctions terminated with iron demonstrate the ohmic type of conductivity. The characteristics are symmetric as a function of polarization voltage and the rectifying ratio, i.e., the ratio of forward to reverse current at maximal voltage, is equal to 1 with accuracy better then ±1%. In contrary the FAT junction has a strongly nonlinear *I*-*V* characteristic (see Figure 7g). For the bipolar junction of semiconducting metal-oxide with a double Schottky diode the nonlinear, but symmetric with polarization voltage current-voltage dependence is usually observed, as found for FAT sample. The rectifying ratio in this case is slightly above 1.1 at 300 K and reduces to 1.02 at 5 K. The shape of the *I*-*V* curve for FAT junction is characteristic for varistor of a two-terminal bipolar diode, where $I=R{\left(V\right)}^{\alpha }*V$ [50,51] with α being the nonlinearity coefficient. The stronger variation of α from unity the stronger deviation from ohmic-type of conductivity. The obtained value of the nonlinearity coefficient $\alpha =\frac{R}{R_{diff}}=\frac{\left(V/I\right)}{{\left(dI/dV\right)}^{-1}}$ [52] in case of FAT junction is around 1.5(1) at 300 K and increases to 2.0(1) at 5 K and does not show variation with magnetic field or measurements geometry. The switching voltage between passive and active state, i.e., a voltage for which a strong deviation from linear *I*-*V* dependence occurs, is around 0.6 V. Similar values of nonlinearity coefficient and switching voltage were found for iron titanites, such as mixtures of hematite-ilmenite with semiconducting properties [51,53]. Therefore, it can be expected that the varistor-like properties in FAT sample might be a result of the intermixing and formation of Fe-Ti-O oxide barrier at the interface between AFeO and top Ti layer. The effect of atomic intermixing in our junctions was previously identified in XRD studies. The values of the nonlinearity factor are relatively small as compared with the most effective varistors based on ZnO or SnO${}_{2}$ ceramics where it can reach values of several dozen or above hundred [54,55]. However, in these materials, the switching voltage is very large, and the active state is obtained for gating with several or more volts, an order of magnitude more then in case of FAT junction.

After careful analysis of the nonlinearity coefficient α and, in particular, the differential resistance $R_{diff}\left(V\right)={\left(\frac{dI}{dV}\right)}^{-1}$ (calculated from *I*-*V* characteristics), a presence of weak deviation from linearity and, hence, from ohmic conductivity has been found in all junctions. The differential resistance is a quantity often used in electronics to observe and characterise the non-ohmic materials [56]. Results of normalized values of voltage-dependent differential resistance, the $VR=\frac{R_{diff}\left(V\right)-R_{diff}\left(V=0\right)}{R_{diff}\left(V=0\right)}$, are presented in Figure 7, together with determined magnetoresistance values MR.

The comparison of MR and VR dependencies shows distinct differences between samples. The junctions with Fe as a top layer (FAF and TAF) show strong MR and weak VR effects while FAT sample exhibits opposite trend. This suggests that the transport properties are governed by the properties of the top metallic layer and the interface between this top layer and anodized oxide. For iron used as the top layer, the influence of the magnetic field on transport properties is strong, and the magnetoresistance effect is large. On the contrary, for the ‘non-magnetic’ titanium layer, the influence of the electric field becomes more evident, and the semiconducting properties of the oxide govern the conducting of the junction and induce a presence of a Schottky-like barrier.

Furthermore, the FAF and TAF junctions show the change of sign for MR and VR parameters from positive to negative with lowering of temperature. At room temperature, both parameters have positive values, while, at low temperatures, they become negative. The negative values of VR seen in Figure 7 are the result of normalization procedure, and no negative differential resistance was obtained for our samples. The positive value of magnetoresistance is a result of Lorentz force acting on electrons leading to a parabolic shape of MR known as ordinary magnetoresistance of metals [57,58]. At low temperature, the negative anisotropic MR is strong in the TAF sample and dominates for the whole range of magnetic fields, while, for the FAF sample, it is weaker and turns into positive parabolic dependence after reaching magnetic saturation. Additionally, in both junctions, either a lack, or a weak dependence of measurements geometry on MR and VR was found. The VR response is very weak with a similar magnitude at both low and high temperature. This suggests that the electric barrier formed at the interface between the iron layer and both anodized oxides shows a weak semiconducting character.

The negative VR and MR dependencies in FAT are demonstrated in Figure 7h,i, respectively. The VR reaches −80%, while the MR is −0.5%. The VR dependence becomes flat for voltage larger than ±0.6 V, i.e., when the switching voltage for varistor is achieved. The MR demonstrates the parabolic-like nature in the range of magnetic field of 0 and ±15 kOe, and for larger field its value becomes independent of magnetic field. The value of ∼15 kOe is comparable with the saturation field found in magnetization studies suggesting that the change in behavior of magnetoresistivity is connected with reorientation and alignment of magnetic moments in the AFeO and bottom Fe layer of the junction. The negative value of MR can result from the presence of giant magnetoresistance (GMR) of granular samples or domain wall resistance (DWR). The DWR arising from a scattering of charge carriers at the boundaries of the magnetic domains was previously identified in iron [59,60], while the presence of GMR was found in magnetite nanoparticles [61] and explained as a gradual relative alignment of magnetic moments between neighboring particles. The anisotropic magnetoresistance effect (AMR) is another possible explanation for the appearance of negative MR. In AMR, a strong dependence of relative direction of current and magnetization vector on the resistivity value is expected [62]. In this case, the magnetoresistance should show a change of sign from positive for longitudinal to negative for transverse geometry, an effect not present in our samples. The effect of MR sign change was previously observed in iron thin films [63]. A small reduction of MR amplitude for different measurement geometry found in FAT sample could indicate that the AMR might be present in this junction, but its importance is minor. Therefore, all three effects, the GMR, DWR, and AMR, can contribute to the observed total negative MR effect, but they cannot be unambiguously identified.

**Figure 8:**
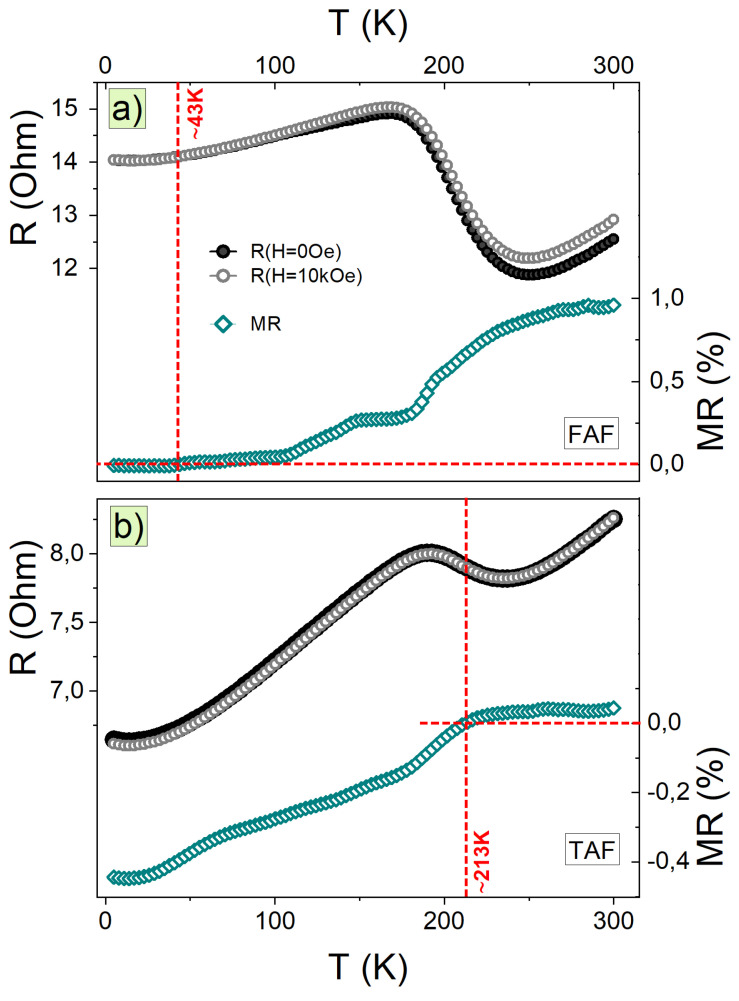
Temperature dependent resistivity R(T) measured without and with a magnetic field of 10 kOe and corresponding magnetoresistance values for (**a**) FAF and (**b**) TAF junctions.

Additionally, we measured the MR(T) curves of FAF and TAF junctions to better understand processes related to sign changes of magnetoresistance. Figure 8 shows temperature dependent resistivity collected without and with magnetic field of 10 kOe and determined values of MR. The temperatures at which MR changes sign are marked with red lines in Figure 8. The change of MR sign was found at 43(1) K for FAF and at 213(1) K for TAF samples.

The effect of magnetoresistance inversion with temperature was previously reported for magnetite thin film and iron epitaxial thin films by Yoon and Hong [64] and Granberg et al. [63], respectively. In magnetite film, the inversion was observed at 264 K. This temperature is higher than our findings for TAF and FAF junction, but, since the magnetite phase was found in all our samples, we can assume that its presence can be responsible for observed MR behavior. Yoon and Hong attributed the phenomena of MR sign reversing to the DWR effect and the scattering of electrons leading to their temperature-dependent spin-flip inside the domain wall. This effect increases the spin diffusion length and decreases the domain wall width with lowering temperature. Such behavior can lead to the change of MR sign at transition temperature [65]. In case of porous samples prepared with the anodization process, we observed a mixture of oxides which induces phase homogeneity and chemical disorder. A diffusion length can be strongly reduced by these factors, leading to the decrease of the transition temperature.

Granberg et al., on the other hand, considered two distinct contributions to the change of magnetoresistance sign with temperature for longitudinal geometry in Fe(001) single crystal thin film. The first negative contribution comes from Lorentz force acting on conduction carriers in ferromagnet. The second component, the extraordinary *MR* effect, arises from spin-orbit coupling and strongly depends on the relative direction of the external magnetic field and the direction of the current within the single magnetic domain which brought a positive MR for single crystalline iron. Therefore, the observed magnetoresistance of Fe thin film is an effect of the competition of ordinary and extraordinary effects. If the temperature is changed, the scattering length of both components change in a different way, leading to an MR sign inversion. Granberg et al. determine the temperature of MR sign reversal being approximately 70 K, which is close to FAF junction inversion temperature. Additionally, they observed strong dependence of inversion temperature on measurement geometry and film thickness. Their result showed that only in samples with a thickness larger than ∼30 nm the MR sign change with temperature is observed from positive to negative values if measured in longitudinal geometry, as opposed to the sample measured in transverse geometry with negative to positive MR sign change. In our case of polycrystalline porous samples, the MR exhibits no measurement geometry dependence, indicating that the scattering at the grain boundaries or interface of oxide and top metal layer is dominant.

## 4. Conclusions

In this paper, we showed that the electrochemical anodization process can be used as an easy way for oxidation of metallic layers and formation of magnetic metal/porous oxide junctions. We studied iron and titanium compounds and prepared metal/metal oxide/metal junctions with different combination of elements. The anodization conditions, such as time and voltage, were chosen in such a way that approximately half of the metal layer was oxidized creating 300 nm thick porous oxide layer. The XRD analysis allowed us to identify various phases of metallic oxides confirming high inhomogeneity of the samples. The atomic intermixing at the interface between oxide and metallic layer was observed, an effect well-pronounced for mixed TAF or FAT junctions where magnetite and hematite or rutile were found, respectively. The chemical inhomogeneities and structural defects influence the magnetic and electrical transport properties. In particular, a large switching field distribution and non-uniform magnetization reversal mechanism were found. Detailed analysis of differential resistivity proved a presence of non-linear current-voltage dependence. The FAT junction showed strong negative VR, independently on the geometry and temperature of measurements. In addition, the MR for this sample had only negative values. On the contrary, for FAF and TAF junctions, we observed the presence of change of VR sign from positive to negative with lowering temperature. Similar behavior was found for magnetoresistance, for which we observed the competition between ordinary MR and combination of DWR, GMR, or AMR phenomena. This competition led to the temperature dependent change of MR sign: at high *T*, the MR sign was positive, while, at low *T*, a negative value was observed. The mutual correlation of the VR and MR signs indicates that the scattering of the charge carriers and, hence, the magnetic or electric field dependence of conductivity properties have the same origin, related to the energy barrier at the metal/oxide interface, porous structure, and chemical inhomogeneity of the samples. The important finding of this study is the effect of the top metallic layer, whether it is a ferromagnetic iron or paramagnetic titanium, and the barrier formed at the interface between this layer and the oxide.

## Figures and Tables

**Table 1:** List of samples.

Sample Labeling	Si/Ti Adhesion Layer/Bottom Au Electrical Contact/ Junction/Top Au Electrical Contact
**FAF**	Si/Ti${}_{50\mathrm{nm}}$/Au${}_{100\mathrm{nm}}$/**Fe/AFeO/Fe**${}_{50\mathrm{nm}}$/Au${}_{50\mathrm{nm}}$
**FAT**	Si/Ti${}_{50\mathrm{nm}}$/Au${}_{100\mathrm{nm}}$/**Fe/AFeO/Ti**${}_{50\mathrm{nm}}$/Au${}_{50\mathrm{nm}}$
**TAF**	Si/Ti${}_{50\mathrm{nm}}$/Au${}_{100\mathrm{nm}}$/**Ti/ATiO/Fe**${}_{50\mathrm{nm}}$/Au${}_{50\mathrm{nm}}$

## Data Availability

The datasets generated and analysed during the current study are available from the corresponding author on reasonable request.

## Appendix A

**Table A1:** Values of coercive field ($H_{C}$), switching field ($H_{SF}$) and its width and percentage area for *H*‖*S* and *H*⊥*S* measured at low and high temperature.

***H*‖*S*** **Geometry**	**10 K**				**300 K**			
	$H_{C}$ **(kOe)**	**Area (%)**	$H_{\mathrm{SF}}$ **(kOe)**	**Width (kOe)**	$H_{C}$ **(kOe)**	**Area (%)**	$H_{\mathrm{SF}}$ **(kOe)**	**Width (kOe)**
**FAF**	0.16(1)	32(1)	0.15(1)	0.13(1)	0.07(1)	34(1)	0.07(1)	0.04(1)
		68(1)	0.15(1)	0.69(1)		66(1)	0.09(1)	0.21(1)
**TAF**	0.16(1)	58(1)	0.14(1)	0.03(1)	0.08(1)	56(1)	0.08(1)	0.02(1)
		42(1)	0.21(1)	0.16(1)		44(1)	0.11(1)	0.22(1)
**FAT**	0.16(1)	100	0.16(1)	0.03(1)	0.12(1)	100	0.128(1)	0.0(1)
***H***‖***S*****Geometry**	**10 K**				**300 K**			
	$H_{C}$ **(kOe)**	**Area (%)**	$H_{\mathrm{SF}}$ **(kOe)**	**Width (kOe)**	$H_{C}$ **(kOe)**	**Area (%)**	$H_{\mathrm{SF}}$ **(kOe)**	**Width (kOe)**
**FAF**	0.33(1)	99(1)	∼0	24.25(4)	0.44(1)	95(1)	∼0	22.25(2)
		1(1)	0.46(1)	2.56(4)		5(1)	0.70(1)	1.33(2)
**TAF**	0.79(1)	92(1)	∼0	23.45(5)	0.65(1)	91(1)	∼0	22.39(4)
		3(1)	1.82(1)	0.29(1)		9(1)	0.96(1)	0.67(1)
		5(1)	1.82(1)	5.52(1)				
**FAT**	0.68(1)	85	∼0	26.11(3)	0.82(1)	94(1)	∼0	25.58(1)
		6(1)	2.17(1)	0.51(1)		6(1)	1.17(1)	0.04(1)
		9(1)	0.11(1)	5.53(1)				
